# Association between metabolic syndrome and bone fracture risk: A community-based study using a fracture risk assessment tool

**DOI:** 10.1097/MD.0000000000009180

**Published:** 2017-12-15

**Authors:** Chia-Ying Yu, Fang-Ping Chen, Li-Wei Chen, Sheng-Fong Kuo, Rong-Nan Chien

**Affiliations:** a Department of Gastroenterology and Hepatology; b Community Medicine Research Center; c Department of Obstetrics and Gynecology; d Metabolism and Endocrinology, Chang-Gung Memorial Hospital and University, Keelung, Taiwan.

**Keywords:** adiponectin, bone fracture, leptin, metabolic syndrome, osteoporosis, tumor necrosis factor

## Abstract

Osteoporosis and metabolic syndrome (MS) share similar risk factors. Previous studies of association between bone marrow density (BMD) and MS are controversial. Moreover, some studies revealed that MS is associated with BMD but not with bone fracture. In clinical practice, patients pay more attention to bone fracture risk than BMD values. Hence, this study aimed to evaluate the association between MS and the 10-year bone fracture risk probability using a fracture risk assessment tool (FRAX) from community-based data. From March 2014 to August 2015, 2689 participants (897 men and 1792 women) were enrolled in this study. Inflammatory cytokines, such as tumor necrosis factor alpha and C-reactive protein, and adipokines were included for analysis.

The mean age was 60.2 ± 10.7 years in men and 58.9 ± 9.6 years in women. The percentage of MS was 27.6% in men and 27.9% in women. Participants were divided into 2 groups, those with or without MS. Compared with women without MS, women with MS had a higher rate of fracture risk (22.8% vs 16.3%, *P* = .001). In contrast, men with MS had a lower rate of fracture risk then men without MS (5.6% vs 12.3%, *P* = .004). However, MS loss the association with a high bone fracture risk in men based on multivariate logistical regression analysis, after adjusting for confounding factor of body mass index (BMI). Conclusively, the result of regression analysis between MS and the bone fracture risk may be different in men and women, and BMI was an important confounding factor to interfere with the regression analysis.

## 1 Introduction

Osteoporosis is defined as decreased bone mass and increased probability of bone fracture, especially in elderly patients.^[1]^ Osteoporotic fractures are associated with higher debility and poor quality of life.^[1]^ The standard method for diagnosing osteoporosis is bone mineral density (BMD) measured by dual-energy x-ray absorptiometry (DXA).^[2]^ However, DXA may not be practical for large-scale surveys such as community screening of bone fracture risk. A fracture risk assessment tool (FRAX) was therefore developed based on the use of clinical risk factors to evaluate the probability of 10-year fracture risk in people 40 to 90 years old.^[3]^ The advantage of FRAX is that bone fracture risk can be evaluated without BMD information, which could therefore make this method applicable for community mass screenings.^[3–5]^ The risk factors for osteoporotic fractures include old age, previous fractures, current tobacco smoking, daily alcohol consumption, long-term use of glucocorticoids, rheumatoid arthritis, parental fracture history, and lower BMD of the femoral neck.^[3]^

Osteoporosis is frequently associated with some risk factors of metabolic syndrome (MS),which include high blood pressure, hyperlipidemia (high levels of triglycerides, low levels of high density cholesterol), central obesity (increased waist girth), and insulin resistance (IR) (high levels of fasting blood sugar).^[6]^ Central obesity and IR are the key pathological hallmarks of MS. Past studies have revealed that osteoporosis is associated with central adiposity, and a patient's glycemic levels predict bone loss and osteoporotic fractures.^[7–9]^ Some inflammatory cytokines and adipokines are related to obesity and IR, such as interleukin-1 (IL-1), tumor necrosis factor alpha (TNF-α), C-reactive protein (CRP), adiponectin, and leptin.^[10,11]^ IL-1 and TNF-α have been reported to increase bone resorption,^[10,12,13]^ and CRP is a systemic inflammatory marker regulated by cytokines such as IL-1, IL-6, and TNF-α.^[12–14]^ Adiponectin and leptin were also reported to be associated with bone resorption.^[15–18]^ Hence, patients with MS may be in a constant state of chronic inflammation and obesity, which may affect osteoporosis and bone fracture risk. Several studies have evaluated the association between MS and osteoporotic fractures, but the results are controversial.^[19–23]^

We hypothesize that patients with MS are in a state of chronic inflammation with increased levels of inflammatory cytokines and adipokines, which therefore results in a higher bone fracture risk. This study aimed to analyze the association between MS and bone fracture risk by FRAX using community-based data. Levels of inflammatory cytokines and adipokines such as TNF-α, CRP, adiponectin, and leptin were also analyzed.

## 2 Methods

### 2.1 Patients

From March 2014 to August 2015, an MS survey was performed in the northeastern region of Taiwan, where 3 communities (Wanli, Ruifang, and Anle) were included. The borough chief would inform the villagers before each survey activity and enroll the participants in the community center. All subjects that joined the examination volunteered. Because MS is mostly diagnosed in middle-aged people or older (>40 years-old), and because of ethical considerations, inclusion criteria were people 40 years or above and not currently pregnant. Exclusion criteria were patients with glucocorticoid usage, use of medication for osteoporosis, currently receiving hormone replacement therapy or oral 25-OH vitamin D3/calcium supplementation, and a history of fractures.

All individuals answered a demographic survey and underwent a complete physical examination and chemical analysis of blood. The demographic survey assessed the history of systemic diseases, medication history, and family history. Physical examination included measurement of basic vital signs, body weight, body height, and waist girth (circumference). Body mass index was calculated as weight (kg) divided by squared height (m). Blood samples were obtained from participants after an overnight fast, and the following parameters were determined: complete blood cell count, liver and renal biochemistry parameters, lipid profiles, fasting plasma glucose and insulin, adiponectin, leptin, TNF-α, and CRP levels. The Institutional Review Board of the Chang-Gung Memorial Hospital approved this research (IRB No. 102–2827C and 103–2392C). All participants agreed to the study conditions and provided informed consent before enrollment.

### 2.2 Metabolic syndrome

A race-specific waist girth threshold based on the NCEPATP III criteria was utilized to prevent distortions in MS prevalence.^[6]^ The cutoff values for normal waist circumference in Asian men and women were set to 90 cm (35 in) and 80 cm (31.5 in), respectively. MS was defined according to the ATPIII criteria as the presence of at least 3 of the following 5 traits: visceral (abdominal) obesity based on waist circumference; blood pressure >130/85 mm Hg or drug treatment for essential hypertension; serum high-density lipoprotein cholesterol (HDL-C) levels <40 mg/dL (1 mmol/L) in men and <50 mg/dL (1.3 mmol/L) in women or drug treatment for low HDL-C; serum triglycerides (TG) level >150 mg/dL (1.7 mmol/L) or drug treatment for elevated TG; and fasting plasma glucose levels >100 mg/dL (5.6 mmol/L) or drug treatment for diabetes mellitus (DM).

### 2.3 Bone fracture risk assessment using the FRAX tool

The FRAX tool without BMD was applied for bone fracture risk assessment (10-year probability of hip or other major osteoporotic fracture). FRAX is based on the assessment of clinical risk factors including age, body weight, tobacco use, excessive alcohol consumption, individual and family history of fracture, glucocorticoid use, secondary osteoporosis, and rheumatoid arthritis. There is a consensus that people with FRAX evaluation of 10-year probability of hip fracture ≥3% or of 10-year probability of major osteoporotic fracture ≥20% may be considered to have a high risk of bone fracture.^[2]^

### 2.4 Determination of TNF-α

A commercial kit (Immunite 1000 LKNF1, Siemens Medical Solutions Diagnostics, Lanberis, UK) with a quantitative sandwich enzyme immunoassay technique was used according to the manufacturer's instructions.

### 2.5 Determination of adiponectin and leptin

A quantitative sandwich enzyme immunoassay method was used. Commercial kits (Human Total Adiponectin/Acrp30, BioVendor Research and Diagnostic system, Minneapolis, MN; Human Leptin ELISA, Clinical Range, BioVendor Laboratory Medicine, Karasek, Czech Republic) were used according to the manufacturers’ instructions.

### 2.6 Statistical analysis

Mean value ± standard deviations (SD) are shown for continuous variables. Either the chi-square test or Fisher exact test was used for categorical data analysis, as appropriate. All statistical tests were 2-tailed. A *P* value of <.05 was considered to indicate a statistically significant difference. Pearson correlation coefficients, phi correlation coefficient, and Spearman rho were used for numerical, nominal, and ordinal data alternatively. For example, Pearson coefficient was applied for continuous data (such as age, BMI, CRP, TNF-α, adiponectin, and leptin). Phi coefficient was applied for binary category data (such as MS). Spearman rho coefficient was used for ordinary data (such as bone fracture risk). Multivariate logistic regression analysis of factors such as metabolic parameters, inflammation markers, and bone fracture risk were performed after adjusting for potential confounders such as age or BMI. The odds ratio (OR) was recorded as OR plus the 95% confidence interval (95% CI). Statistical analyses were performed using SPSS for Windows (Version 16.0, SPSS, Chicago, IL).

## 3 Results

**Figure 1:**
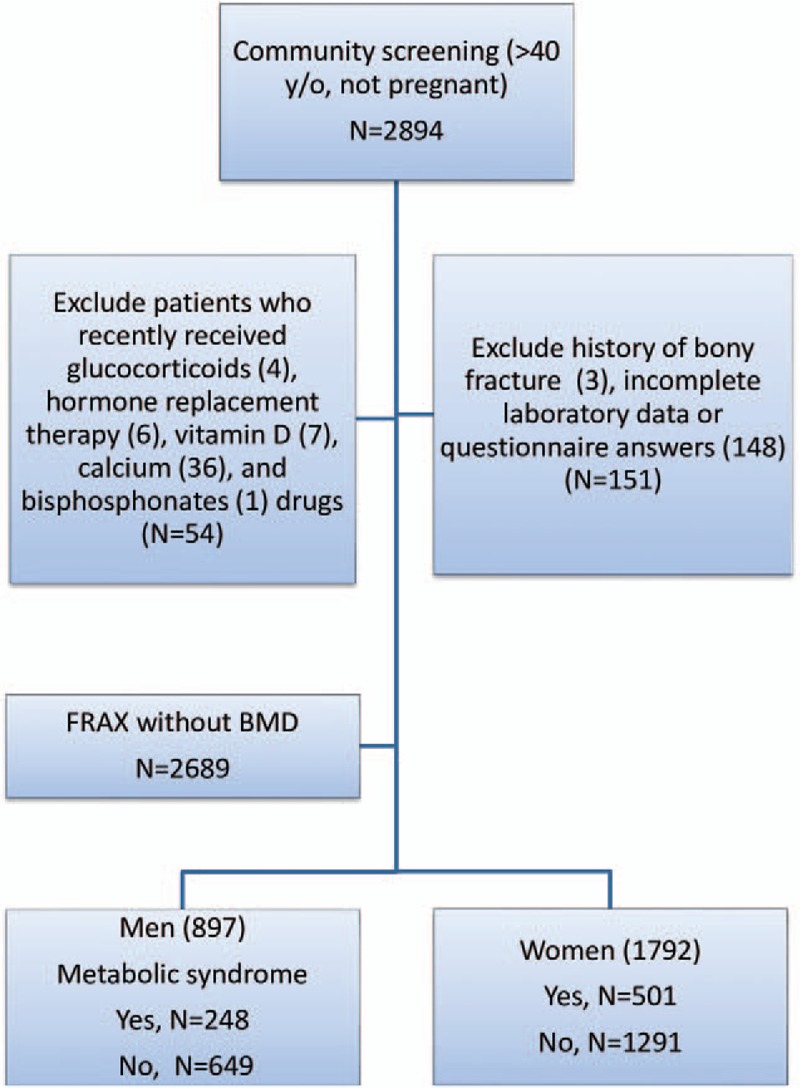
Flow diagram.

Initially, 2894 participants were enrolled. A total of 205 participants were excluded because of drugs interfering with fracture analysis (54), bony fracture history (3), and incomplete laboratory data or questionnaire answers (148). Finally, 2689 participants (897 men and 1792 women) were selected in this study (Fig. 1). The demography is shown in Table 1 (men) and Table 2 (women). The mean age of participants was 60.2 ± 10.7 years and 58.9 ± 9.6 years for men and women, respectively. The frequency of MS was 27.6% in men and 27.9% in women. Participants were divided into 2 groups, with and without MS.

**Table 1:**
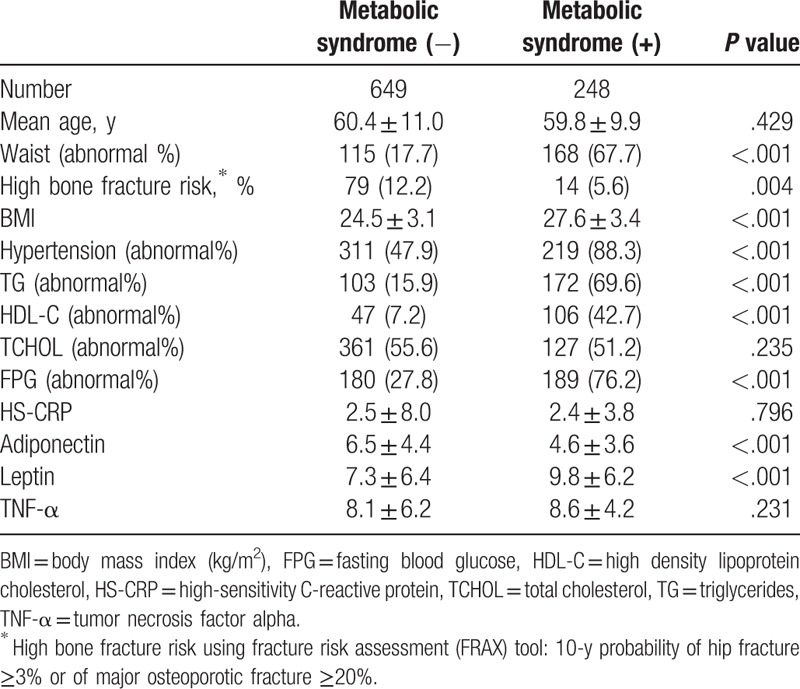
Demography and characteristics of the male group.

**Table 2:**
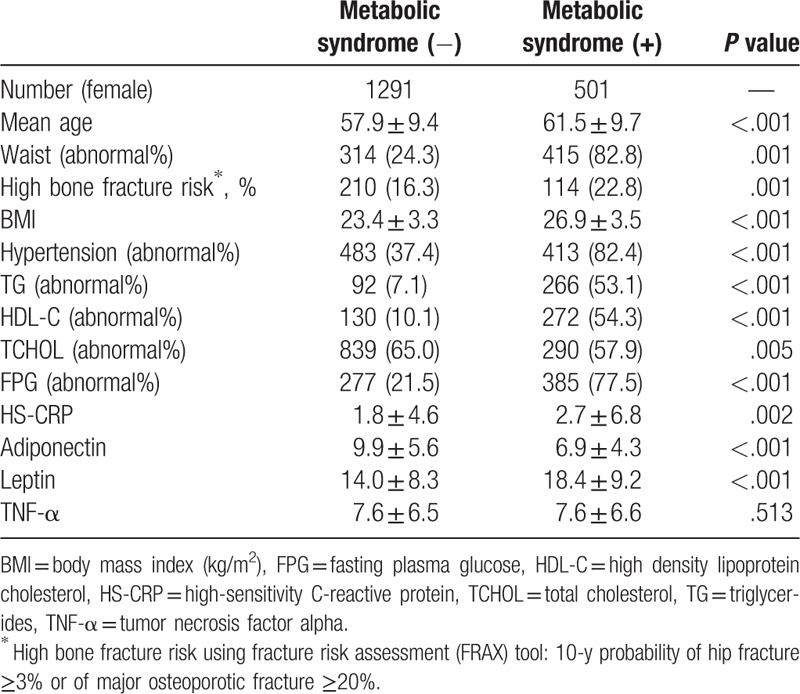
Demography and characteristics of the female group.

A total of 324 women and 93 men were considered at high risk of bone fracture. Compared to men without MS, men with MS had a lower chance of high fracture risk (5.6% vs 12.2%, *P* = .004). Moreover, the levels of adiponectin, leptin, CRP, and TNF-α were significantly different between men with MS and without MS. We found that a high fracture risk negatively correlated with MS and BMI, but positively correlated with age and adiponectin levels (Table 3). In contrast to the results from men group, women with MS had a higher risk of fracture risk than those without MS (22.8% vs 16.3%, *P* *=* .001; Table 2). Similar to the results from the men group, the mean values of adiponectin, leptin, CRP, and TNF-α were significantly different between women with MS and those without MS. Table 3 shows that MS, age, adiponectin, and TNF-α values were positively correlated with high fracture risk.

**Table 3:**
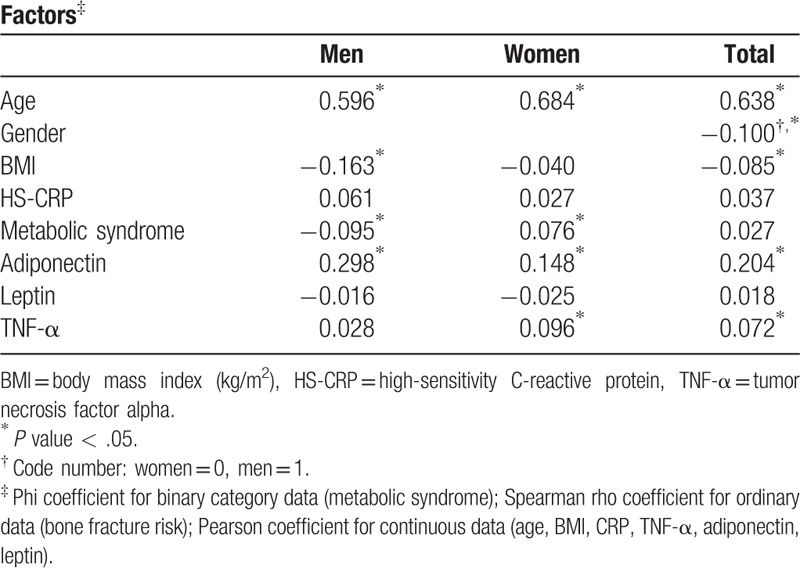
Correlation between high fracture risk and variable factors.

Table 4 shows the results of the association between factors and high bone fracture risk by univariate and multivariate logistic regression analysis. A collinearity diagnostic evaluation was conduct including factors of age, gender, BMI, adiponectin, leptin, TNF-α, HS-CRP, and 5 traits of MS. The result revealed the values of variance inflation factor (VIF) were all <10 (1.018–1.502). Hence, these variables may be not collinearity and could be used for regression analysis. In univariate logistical regression analysis, MS decreased the risk of high bone fracture (OR = 0.432, 95% CI = 0.240–0.777) in men but MS increased the risk of high bone fracture (OR = 1.516, 95% CI = 1.174–1.958) in women. In multivariate logistical regression analysis by adjusting the factors of age, BMI, and adiponectin, MS would loss the association with high bone fracture in men group (OR = 1.126, 95% CI = 0.273–4.650, *P* = .869). In women group, the factors of age, adiponectin, TNF-α, but not BMI were adjusted in the multivariate logistical regression. MS was still positively associated with the risk of bone fracture in women (OR = 0.304, 95% CI = 0.165–0.559). After using the 5 traits of MS as factors of high fracture risk into multivariate logistic regression analysis, the results show that only waist was negatively associated with high fracture risk in men (OR = 0.921, 95% CI = 0.871–0.973, *P* = .003) and in women (OR = 0.868, 95% CI = 0.836–0.901, *P* < .001) after adjusting for age (Table 5).

**Table 4:**
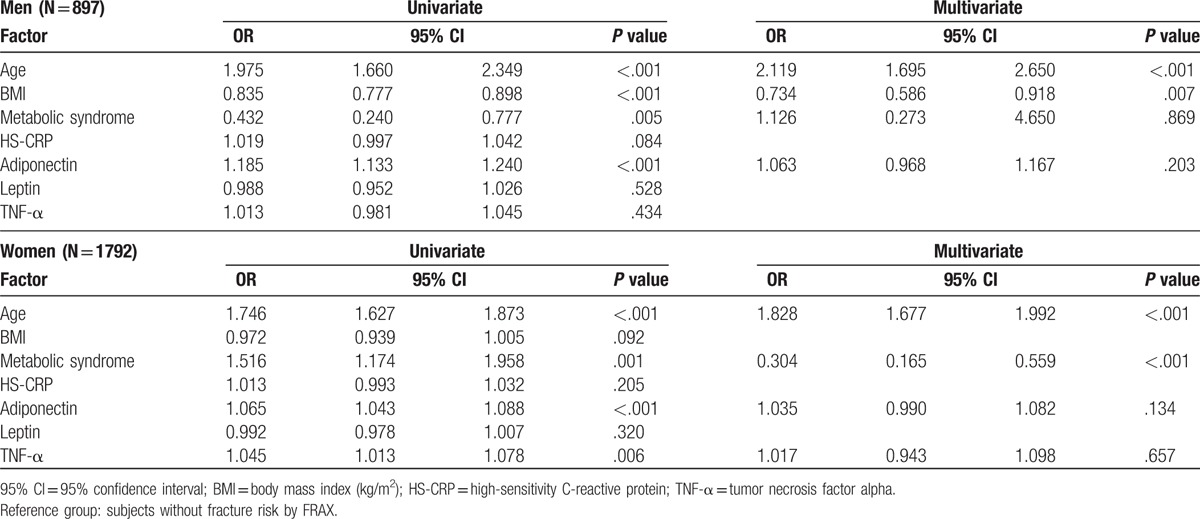
Factors associated with high bone fracture risk by univariate and multivariate logistic regression analysis.

**Table 5:**
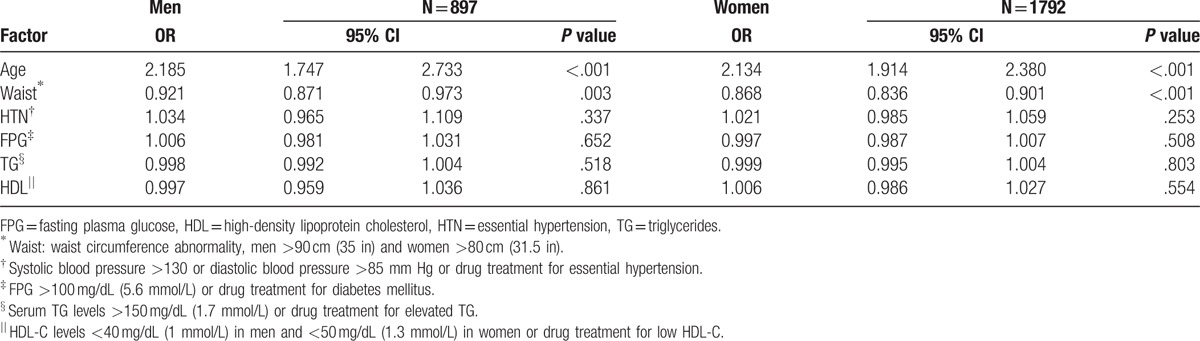
Multivariate logistic regression analysis using components of metabolic syndrome as predictors of high fracture risk.

## 4 Discussion

Many studies evaluated the relationship between osteoporotic fracture and MS, but the results are controversial.^[19–24]^ This inconsistency is probably a result of differences in ethnic background, gender, and study design. Western women with MS seem to have a lower fracture risk than those without MS.^[19]^ In contrast, Eastern women with MS show a higher fracture risk than those without MS.^[20]^ Despite the association observed between MS and bone fracture risk in women, most studies reported no association or negative association between MS and bone fracture risk in men.^[20–22]^ Factors such as larger bone size and strength, shorter life expectancy, and different levels of sex steroids including estradiol and testosterone underlie the different prevalence and incidence of osteoporosis between women and men.^[25,26]^ A cross-sectional study of 9930 Chinese adults over 40 years revealed that the prevalence of osteoporotic fractures was significantly higher in women with MS than those without MS, but this difference was not observed in men.^[20]^ In contrast, French and Japanese men with MS have a lower risk of fracture.^[7,21]^ Our study also revealed that women and men with MS had a different risk for bone fracture. Women with MS had a higher risk (OR = 1.516) for bone fracture than women without MS. However, men with MS had a lower risk (OR = 0.432) for bone fracture than men without MS. When adjusting the factor of BMI, MS lost the association with bone fracture risk in the multivariate regression analysis in men. Hence, BMI was an important confounding factor between MS and high bone fracture risk.

In a Morocco study with 270 post-menopausal women, it was reported that women with MS had a higher BMD at the hip and spine, suggesting a protective effect of MS on bone. However, the prevalence of vertebral fracture was similar between women with or without MS.^[22]^ Although women with MS may have a higher BMD, mainly driven by a larger body weight, other factors such as a worse bone quality and/or a greater tendency to fall may induce a higher fracture risk.^[25]^ In clinical practice, patients might be more concerned with osteoporotic fracture risk than with BMD values. Although BMD is the gold standard to access osteoporosis, this condition is still not the same as bone fracture. Thus, it is difficult to interpret, as reported in some studies, the association between MS and BMD without association between MS and osteoporotic fractures.^[22,23]^ FRAX provides an easier and straightforward method to predict facture risk in clinical practice.

The main MS factors, high blood pressure, hyperlipidemia, or hyperglycemia, may have an influence on bone fracture or BMD. In a Korean population-based study of 3207 subjects, the triglyceride component of MS was reported as related to lower femoral head BMD, whereas hyperglycemia was related to a higher total hip BMD in men.^[27]^ A 3-year retrospective longitudinal study conducted in Korea of 1218 postmenopausal women showed that differences in annualized BMD changes related to MS status were not observed when adjusting for confounding factors such as weight and height.^[28]^ In our study, waist circumference (central obesity) was the most important component of MS associated with a high fracture risk. In both sexes, a negative association with waist circumference for a high fracture risk was seen in multivariate regression analysis.

In terms of inflammatory cytokines, CRP levels have been shown to share a significant association with non-traumatic fracture.^[13]^ A study by Koh et al showed that CRP levels greater than 1.2 and 1.8 mg/L for premenopausal and postmenopausal women, respectively, were significantly associated with osteoporosis.^[14]^ In our study, the mean value of CRP was higher in male participants with a risk of bone fractures. In women, TNF-α level was higher in participants with a high bone fracture risk. However, the association of CRP and TNF-α levels with bone fracture risk was not observed after adjusting for either age or BMI. Indeed, BMI is clearly the most important factor associated with bone fracture risk in our study.

The present study has some limitations. First, we did not perform DXA measurements to access bone fracture risk. Our study was a community-based survey looking at the association between MS and bone fracture risk. DXA may not be applicable as a mass screening tool. The FRAX tool was used to estimate the 10-year risk of major osteoporotic or hip fracture based on clinical factors alone, but the actual prevalence of bone fracture will have to be determined with follow-up studies. Second, this was a community-based and cross-sectional study. A selection bias may therefore exist and the results may not extend to other populations, a common flaw in cross-sectional studies. Further longitudinal cohort studies are warranted for confirming the role of MS, adipokines, and inflammatory cytokines in bone fracture development.

In conclusion, men with MS had a negative association with bone fracture, whereas, in contrast, women with MS had a positive association as assessed by FRAX. BMI was an important confounding factor between MS and high bone fracture risk in regression analysis.
